# Study of the fetal and maternal microbiota in pregnant women with intrauterine growth restriction and its relationship with inflammatory biomarkers: A case-control study protocol (SPIRIT compliant)

**DOI:** 10.1097/MD.0000000000022722

**Published:** 2020-11-13

**Authors:** Sergi Fernandez-Gonzalez, Olimpia Ortiz-Arrabal, Ariadna Torrecillas, Miriam Pérez-Cruz, Natalia Chueca, María D. Gómez-Roig, Carolina Gómez-Llorente

**Affiliations:** a Hospital de Sant Joan de Déu. D’Esplugues de Llobregat, Passeig Sant Joan de Déu 2. Esplugues, Barcelona; b Biochemistry and Molecular Biology II Department, School of Pharmacy, University of Granada, Campus de Cartuja s/n; c Department of Microbiology, University Hospital Campus de la Salud; d Instituto de Investigación Biosanitaria ibs. GRANADA; e Institute of Nutrition and Food Technology “Jose Mataix”, Center of Biomedical Research, University of Granada, Granada; f CIBEROBN (CIBER Physiopathology of Obesity and Nutrition), Instituto de Salud Carlos III, Madrid, Spain.

**Keywords:** fetal growth restriction, gastrointestinal microbiota, inflammatory biomarkers

## Abstract

In general terms, fetal growth restriction (FGR) is considered the impossibility of achieving the genetically determined potential size. In the vast majority of cases, it is related to uteroplacental insufficiency. Although its origin remains unknown and causes are only known in 30% of cases, it is believed to be related to an interaction of environmental and genetic factors with either a fetal or maternal origin. One hypothesis is that alterations in the gastrointestinal microbiota composition, and thus alteration in the immune response, could play a role in FGR development. We performed an observational, prospective study in a subpopulation affected with FGR to elucidate the implications of this microbiota on the FGR condition.

A total of 63 fetuses with FGR diagnosed in the third trimester as defined by the Delphi consensus, and 63 fetuses with fetal growth appropriate for gestational age will be recruited. Obstetric and nutritional information will be registered by means of specific questionnaires. We will collect maternal fecal samples between 30 to 36 weeks, intrapartum samples (maternal feces, maternal and cord blood) and postpartum samples (meconium and new-born feces at 6 weeks of life). Samples will be analyzed in the Department of Biochemistry and Molecular Biology II, Nutrition and Food Technology Institute of the University of Granada (UGR), for the determination of the gastrointestinal microbiota composition and its relationship with inflammatory biomarkers.

This study will contribute to a better understanding of the influence of gastrointestinal microbiota and related inflammatory biomarkers in the development of FGR.

Trial registration: NCT04047966. Registered August 7, 2019, during the recruitment stage. Retrospectively registered. Ongoing research.

## Abstract 2

Supplemental Digital Content is available in the text

## 1 Introduction

Fetal growth restriction (FGR) is usually defined as a failure to achieve fetal growth potential and affects up to 6% to 10% of all new-born.^[1–3]^ FGR is associated with poor perinatal and long-term postnatal outcomes, such as worse long-term neurological, cardiovascular, and endocrinological development.^[4–5]^

The current FGR classification differentiates between early- and late-onset FGR. Early-onset cases are the most severe forms with worse outcomes, but late-onset FGR represents 70% to 80% of the FGR, and it is also associated with suboptimal neurological and cardiovascular outcomes. This classification helps in the understanding of the different presentations of the disease, and the cut-off to define each phenotype has commonly been set at approximately 32 to 34 weeks at diagnosis. In addition, a new classification within FGR differentiates 2 populations, that is, intrauterine-growth-restriction (IUGR) and small-for-gestational-age (SGA), according to the presence of several criteria such as growth below the 3rd percentile or abnormal uterine artery Doppler (UtA) or cerebroplacental ratio (CPR). This classification has been demonstrated to be very useful for identifying small fetuses with worse perinatal outcomes and short-term cardiovascular outcomes.^[6]^

Maternal, genetic, and environmental factors such as smoking, hypertension, severe chronic anemia, pregestational diabetes mellitus, autoimmune diseases, genetic alterations, congenital infections or malformations increase the risk of developing FGR, and those risk factors explain 30% of the cases.^[7]^ It is postulated that placental dysfunction may play an important role in this subpopulation.^[8]^ Studies performed in animals and humans have suggested that the microbial colonization process is crucial for immunological and physiological development.^[9]^ The human microbiome is defined as the set of microorganisms that inhabit each part of the body. Until recently, it was accepted that the uterus was a sterile environment, but this concept has changed due to new sequencing techniques that suggest that the fetus, the placenta, and the amniotic fluid are not sterile and that the acquisition and colonization of the gastrointestinal tract begin in the uterus.^[10]^ This fact could also be explained by the meconium, which is the first intestinal discharge of the new-born. In the recent literature, it has been shown to have similar types of microorganisms as those of the amniotic fluid and placenta, reflecting the uterus.^[11]^ Furthermore, the neonatal microbiota is affected by the mode of delivery; however, recent results suggested that within the first 6 weeks of life, the infant microbiota undergoes substantial reorganization that is mainly driven by body site.^[11]^ The alteration of the gastrointestinal microbiota, also called dysbiosis, has been related to a number of diseases, namely, obesity, metabolic syndrome, diabetes, and maternal-fetal conditions.^[12,13]^ The composition of the intestinal microbiota during pregnancy undergoes profound changes, some of which are similar to those observed in obesity.^[14]^ For example, it has been described that there is an increase in the number of intestinal bacteria that potentially metabolize amino acids at the end of pregnancy, which is more abundant in pregnant women who are overweight than in those with normal weight.^[14]^ Other studies indicate that changes in the intestinal microbiota may contribute to the development of pre-eclampsia,^[15]^ and the alteration of the gastrointestinal and vaginal microbiota has been related to an increased risk of preterm birth.^[16]^ Despite the current interest in this issue, there are no concrete data on the relationship between maternal and fetal intestinal microbiota and FGR.

It is well known that the gastrointestinal microbiota is an essential mediator of metabolism and the immune system. There is strong evidence that complications during pregnancy, including FGR, are associated with aberrant maternal inflammation.^[17]^ Similarly, it has been demonstrated in animal models that activation of Toll-like receptor 4 (TLR4) induces premature birth and FGR.^[18]^ In addition, an alteration in the composition of the intestinal microbiota results in high levels of lipopolysaccharide (LPS), which is a component of the Gram-negative bacterial wall and the main ligand of TLR4. The junction of LPS-TLR4 produces a metabolic endotoxemia capable of modulating pro-inflammatory cytokines. In fact, women affected by FGR/pre-eclampsia have a high inflammatory status characterized by an increase in cytokines and chemokines such as tumor necrosis factor alpha (TNF-α) and interleukin (IL) 6, both systemically and in the placenta.^[19]^ Furthermore, it has been demonstrated in animals that maternal exposure to LPS at the end of pregnancy leads to FGR development.^[20]^

According to what has been previously stated, it seems that there is a relationship between the intestinal microbiota and the development of FGR. However, more clinical studies are needed to support this hypothesis. The present study proposes the realization of an observational clinical study in a population with late-onset FGR (pregnant women and new-born), compared to non FGR population (pregnant women and new-borns with fetal growth appropriate for gestational age [AGA]).

The main aim of the study is to identify the modification in the intestinal microbiota in pregnant women and new-borns with pregnancies with FGR in comparison with AGA. Secondary aims are to determine differences in the blood inflammatory biomarkers between pregnant women and new-borns with FGR and AGA controls. We will also assess whether changes in inflammatory parameters are associated whit modification of the intestinal microbiota. Finally, we will also studies the intestinal microbiota of the neonates and controls at 6 weeks of life and to assess its correlation with microbiota composition after birth (meconium).

## 2 Methods

### 2.1 Study design

This study is an observational, prospective study. The trial will be conducted by members of both BCNatal (Barcelona Center of Maternal-Fetal and Neonatal Medicine), Hospital Sant Joan de Déu, University of Barcelona as well as the Institute of Food Nutrition and Technology **“**José Mataix**”**, Department of Biochemistry and Molecular Biology II of the University of Granada. Participants will include 63 pregnant women with fetal growth appropriate for gestational age (AGA) and 63 women affected by FGR, recruited during the third trimester of pregnancy (30–36 weeks). At BCNatal, cases will be recruited as all of the consecutive small fetuses diagnosed during the period of the study, and controls will be randomly selected from low-risk pregnancies. Pregnant women will answer some questionnaires about obstetric, nutritional, demographic, epidemiological, and clinical variables. Maternal samples (feces) will be harvested between 30 to 36 weeks as well as intrapartum samples (feces, maternal, and cord blood) and postpartum samples (meconium and feces of new-borns at 6 weeks of age). In Table 1, we depict the specimen collection schedule. The data and samples will be codified according to the BCNatal – Hospital Sant Joan de Déu biobank and sent to the biobank of the Public Health System of Andalusia (BBSPA). The samples will be sent, managed and processed in accordance with the BBSPA guidelines. All analyses will be performed in the Department of Biochemistry and Molecular Biology II of the University of Granada.

**Table 1:** Maternal and infant specimen collection schedule.

	t0	t1	t3	t4
Timepoint	30-36 weeks of pregnancy	Delivery	24 h after delivery	6 weeks after delivery
*Enrolment:*				
Eligibility screen	X			
Informed consent	X			
Questionnaires recorded	X			
*Maternal Sample collection*:				
Maternal faeces	X	X		
Maternal blood	X	X		
*Cord blood*		X		
*Infant sample collection:*				
Meconium			X	
Faeces				X

The BIOCIR protocol (BIOCIR version 1) has been approved by the local Ethics Committee of Hospital Sant Joan de Déu and Granada with references PIC-40–18 and 02032018, respectively, and will be conducted according to the standards given in the Declaration of Helsinki and Good Clinical Practice Guidelines. Data will be protected from uses not allowed by people outside the investigation, and their confidentiality will be respected in accordance with Organic Law 3/2018, of December 5, on the Protection of Personal Data and Digital Rights and Law 41/2002, of November 14, basic law regulating patient autonomy and rights and obligations regarding information and clinical documentation. All investigators participating in the study are appropriately qualified.

### 2.2 Study population and eligibility criteria

The study population will comprise 126 singleton pregnancies recruited during the third trimester of gestation (30–36 weeks). It will include 63 AGA (fetuses with estimated fetal weight (EFW) > 10th percentile) and 63 fetuses with FGR (EFW <10th percentile), according to the Delphi criteria.^[21]^ In all pregnancies, gestational age will be calculated based on crown-rump length at the first-trimester ultrasound,^[22]^ and estimated fetal and birth weight centiles will be calculated adjusted for the gestational age at delivery and neonatal sex using local reference curves.^[23]^ At the recruitment time, the exclusion criteria will be the following: drug consumption, including tobacco, alcohol ingestion, use of any antibiotics during the 3 months prior to recruitment, premature rupture of membranes, gestational diabetes, and fetus malformation. Furthermore, all pregnant women with delivery before 37 weeks will be excluded. The first patient was recruited on July, 2018 and the estimated recruitment competition date has been extended until December 31, 2020.

The STrengthening the Reporting of Observational studies in Epidemiology (STROBE) Statement guidelines for this observational study and the Standard Protocol Items: Recommendations for interventional trial has been follow-up (Additional file 1).

### 2.3 Sample size

The sample size was estimated based on the primary outcome: intestinal microbiota composition. Based on the results of previous studies,^[15]^ the number of subjects that need to be included in the study to detect differences among the studied cohorts, assuming an alpha error of 5% and a beta error of 20%, is 53 pregnant women per branch. Assuming an acceptance rate of 80%, we need to include a total of 63 pregnant women in each branch in the initial sample.

### 2.4 Outcome measures

#### 2.4.1 Intestinal microbiota composition

The determination of the intestinal microbiota composition will be carried out from 16S rRNA analysis. DNA will be obtained using the QIAamp DNA stool mini kit (Qiagen, Barcelona, Spain) and the PureLink Microbiome DNA purification kit (Invitrogen) from maternal and neonatal fecal samples, and the quality and quantity will be tested spectrophotometrically using a NanoDrop ND-100 spectrophotometer. The variable region V3-V4 of the 16S rRNA gene will be PCR-amplified using specific oligonucleotides.^[24]^ Subsequently, a second amplification will be performed using barcode oligonucleotides. Individual “barcode” sequences are added to each DNA fragment during next-generation library so that each read can be identified and sorted before the final data analysis. These barcodes, or index adapters, are unique identifiers on both ends of the sample. It involves 96 unique Index 1 (i7) adapters and 96 unique Index 2 (i5) adapters, preventing repeated sequences in a well plate (Illumina, Inc). The purified PCR product will be sequenced on an Illumina MiSeq platform, which is located in the Microbiology Service of the San Cecilio University Hospital in Granada, Spain. Estimated completion data on May 2021.

#### 2.4.2 Inflammatory biomarkers

The study of inflammatory biomarkers will be performed in plasma samples from maternal (t0 and t1) and umbilical cord blood (t1). The concentrations of LPS and LBP will be determined by simple ELISA using LS-F17912 and LS-F21929 kits (LSbio), respectively. Simultaneous detection of inflammatory biomarkers will be performed using MILLIPLEXMAP test kits (Millipore Corp, Billerica, MA) and Luminex xMAP detection technology. The HSTCMAG-28SK kit will be used for the determination of IL-6, IL-8, IL-10, IL-23 and IFN-γ and HCYTOMAG-60K for VEGF and IL-15. Estimated completion data on April 2021.

### 2.5 Statistical analysis

Data analysis will be performed at the end of the observational study. The normality of variables will be assessed. Variables not following a normal distribution will be log transformed. Normal variables will be expressed as the mean and standard error of the mean (SEM). In order to determine differences between the case-control group a Students *t* test for independent samples will be carried out. In addition, a one-way ANOVA will be also performed. If the measured variables are not distributed in a normal way, the analysis of the data will be performed by non-parametric tests of comparison of distributions. The Mann–Whitney *U* test will be used for the case of 2 groups. Linear regression models, Principal Components Analysis (PCA) and Pearson Correlations will be used to determine associations of the intestinal microbiota composition with inflammatory biomarkers and clinical parameters. A statistical significance of 0.05 will be established for the contrasts of hypotheses for the population inference. Statistical analyses will be performed using R 3.0 and SPSS v20.0 (IBM, Chicago, IL) programs.

For the analysis of the sequences obtained by parallel massive sequencing, a pre-processing of the sequence analysis based on the MG-RAST server will first be performed. Mothur will be used for denoising, elimination of redundancy and thus making a correct alignment for developing distance matrices, clusters and OTUs (operational taxonomic units). We will calculate the intra-sample diversity (diversity-α) with the obtained OTUs. We will use the Greengenes database to taxonomically classify all sequences. The β diversity will be calculated and thus generate 3D principal coordinate analysis (PCoA) diagrams for representing the differences between the various samples. For the detection of differences taking into account the abundance between groups, we will use “Metastats”.

## 3 Discussion

Our study is a case-control clinical trial that aims to determine the gastrointestinal composition and related inflammatory biomarkers in FGR. Previous studies have described an association between the gastrointestinal microbiota and different maternal-fetal conditions. In this regard, supplementation with specific probiotic strains, namely, *Lactobacillus rhamnosus* GG and *Lactobacillus acidophilus,* together with dietary recommendations, has been found to improve glucose metabolism in pregnant and lactating women.^[25]^ In rats, it has been described that FGR induces changes in the intestinal microbiota composition, and those changes persisted throughout life.^[26]^ However, there is very little knowledge about the interplay between gastrointestinal microbiota, inflammatory biomarkers and FGR in humans. Additionally, in healthy pregnant women has been shown that maternal microbiota can be clustered in 2 distinct microbial groups predominantly charazed by a higher abundance of *Prevotella* (Cluster I) and a higher abundance of the *Rominococcus Kspecies (Cluster II).* This cluster were associated to a diet rich in carbohydrate (Cluster I) or with higher intakes of vegetables proteins and fats (Cluster II).^[27]^

To the best of our knowledge, this is the first study registered on ClinicalTrials.gov with the aim of studying the relationship between the FGR condition and microbiota. Fetal growth is a complex process affected by different factors coming from the mother and/or the fetus. We hypothesize that both the gastrointestinal microbiota and the related inflammatory processes have an important role in the development of FGR. This study will also allow us to understand the underlying mechanisms in this relationship.

There are limitations to this study. First, we are aware that microbiota can be affected by different factors (genetic, stress, diet, physical activity, and delivery mode, among others), and for this reason, we will also collect lifestyle questionnaires. Besides, 1 important issue is the homogeneity of the patients, for that we established a stringent set of inclusion and exclusion criteria in an attempt to control this factor.

All things considered, the obtained results will help to better understand the FGR pathophysiology and the related mechanisms, and the results of this study could be translated into new clinical recommendations to improve both maternal and fetal health.

## Acknowledgments

This paper will be part of Sergi Fernández Gonzalez's doctorate, which is being completed at the Universitat of Barcelona, Spain. RETICS funded by the PN I+D+D 2012–2015 (SPAIN), ISCIII-SUB-Directorate General for Research Assessment and Promotion and the European Regional (FEDER) REF. RD16/0022/000.

## Author contributions

**Conceptualization:** Miriam Pérez-Cruz, M Dolores Gómez-Roig, Carolina Gomez Llorente.

**Investigation:** Sergi Fernandez-Gonzalez, Ariadna Torrecillas, Natalia Chueca.

**Supervision:** Gómez-Llorente C.

**Writing – original draft:** Olimpia Ortiz-Arrabal, Carolina Gomez Llorente.

**Writing – review & editing:** Sergi Fernandez-Gonzalez, Miriam Pérez-Cruz, M Dolores Gómez-Roig.

## Supplementary Material

Supplemental Digital Content

## References

[1] M. Kady S Gardosi J Perinatal mortality and fetal growth restriction. Best Pract Res Clin Obstet Gynaecol 2004;18:397–410. 15183135 10.1016/j.bpobgyn.2004.02.009

[2] Jarvis S Glinianaia SV Torrioli M-G Cerebral palsy and intrauterine growth in single births: European collaborative study. Lancet 2003;362:1106–11. 14550698 10.1016/S0140-6736(03)14466-2

[3] Barker DJP Godfrey KM Gluckman PD Fetal nutrition and cardiovascular disease in adult life. Lancet 1993;341:938–41. 8096277 10.1016/0140-6736(93)91224-a

[4] Wienerroither H Steiner H Tomaselli J Intrauterine blood flow and long-term intellectual, neurologic, and social development. Obstet Gynecol 2001;97:449–53. 11239655 10.1016/s0029-7844(00)01158-3

[5] Hecher K From the fetus at risk to intelligence, educational attainment and psychological distress in the young adult. Ultrasound Obstet Gynecol 2007;29:612–3. 17523159 10.1002/uog.4050

[6] Figueras F Gratacós E Update on the diagnosis and classification of fetal growth restriction and proposal of a stage-based management protocol. Fetal Diagn Ther 2014;36:86–98. 24457811 10.1159/000357592

[7] Kalanithi LEG Illuzzi JL Nossov VB Intrauterine growth restriction and placental location. J Ultrasound Med 2007;26:1481–9. 17957042 10.7863/jum.2007.26.11.1481

[8] Suhag A Berghella V Intrauterine growth restriction (IUGR): etiology and diagnosis. Curr Obstet Gynecol Rep 2013;2:102–11.

[9] Koleva PT Kim J-S Scott JA Microbial programming of health and disease starts during fetal life. Birth Defects Res Part C Embryo Today Rev 2015;105:265–77. 10.1002/bdrc.21117 26663884

[10] Collado MC Rautava S Aakko J Human gut colonisation may be initiated in utero by distinct microbial communities in the placenta and amniotic fluid. Sci Rep 2016;6:23129. 27001291 10.1038/srep23129 PMC4802384

[11] Chu DM Ma J Prince AL Maturation of the infant microbiome community structure and function across multiple body sites and in relation to mode of delivery. Nat Med 2017;23:314–26. 28112736 10.1038/nm.4272 PMC5345907

[12] Qin J Li Y Cai Z A metagenome-wide association study of gut microbiota in type 2 diabetes. Nature 2012;490:55–60. 23023125 10.1038/nature11450

[13] Morgan XC Tickle TL Sokol H Dysfunction of the intestinal microbiome in inflammatory bowel disease and treatment. Genome Biol 2012; 13:R79. 10.1186/gb-2012-13-9-r79 PMC3506950 23013615

[14] Koren O Goodrich JK Cullender TC Host remodeling of the gut microbiome and metabolic changes during pregnancy. Cell 2012;150:470–80. 22863002 10.1016/j.cell.2012.07.008 PMC3505857

[15] Amarasekara R Jayasekara RW Senanayake H Microbiome of the placenta in pre-eclampsia supports the role of bacteria in the multifactorial cause of pre-eclampsia. J Obstet Gynaecol Res 2015;41:662–9. 25492799 10.1111/jog.12619

[16] Witkin SS The vaginal microbiome, vaginal anti-microbial defence mechanisms and the clinical challenge of reducing infection-related preterm birth. BJOG 2015;122:213–8. 25316066 10.1111/1471-0528.13115

[17] Cotechini T Komisarenko M Sperou A Inflammation in rat pregnancy inhibits spiral artery remodeling leading to fetal growth restriction and features of preeclampsia. J Exp Med 2014;211:165–79. 24395887 10.1084/jem.20130295 PMC3892976

[18] Zhao M Chen Y-H Dong X-T Folic acid protects against lipopolysaccharide-induced preterm delivery and intrauterine growth restriction through its anti-inflammatory effect in mice. PLoS One 2013;8:e82713. 24324824 10.1371/journal.pone.0082713 PMC3855776

[19] Szarka A Rigó J Lázár L Circulating cytokines, chemokines and adhesion molecules in normal pregnancy and preeclampsia determined by multiplex suspension array. BMC Immunol 2010;11:59. 21126355 10.1186/1471-2172-11-59 PMC3014878

[20] Xu D-X Chen Y-H Wang H Effect of N-acetylcysteine on lipopolysaccharide-induced intra-uterine fetal death and intra-uterine growth retardation in mice. Toxicol Sci 2005;88:525–33. 16162852 10.1093/toxsci/kfi300

[21] Gordijn SJ Beune IM Thilaganathan B Consensus definition of fetal growth restriction: a Delphi procedure. Ultrasound obstet gynecol 2016;48:333–9. 26909664 10.1002/uog.15884

[22] Robinson HP Sweet EM Adam AH The accuracy of radiological estimates of gestational age using early fetal crown-rump length measurements by ultrasound as a basis for comparison. Br J Obstet Gynaecol 1979;86:525–8. 476017 10.1111/j.1471-0528.1979.tb10804.x

[23] Figueras F Meler E Iraola A Customized birthweight standards for a Spanish population. Eur J Obstet Gynecol Reprod Biol 2008;136:20–4. 17287065 10.1016/j.ejogrb.2006.12.015

[24] Klindworth A Pruesse E Schweer T Evaluation of general 16S ribosomal RNA gene PCR primers for classical and next-generation sequencing-based diversity studies. Nucleic Acids Res 2013;41:e1. 22933715 10.1093/nar/gks808 PMC3592464

[25] Brantsaeter AL Myhre R Haugen M Intake of probiotic food and risk of preeclampsia in primiparous women: the Norwegian Mother and Child Cohort Study. Am J Epidemiol 2011;174:807–15. 21821542 10.1093/aje/kwr168 PMC3203379

[26] Fança-Berthon P Hoebler C Mouzet E Intrauterine growth restriction not only modifies the cecocolonic microbiota in neonatal rats but also affects its activity in young adult rats. J Pediatr Gastroenterol Nutr 2010;51:402–13. 20601908 10.1097/MPG.0b013e3181d75d52

[27] García-Mantrana I Selma-Royo M González S Distinct maternal microbiota clusters are associated with diet during pregnancy: impact on neonatal microbiota and infant growth during the first month of life. Gut Microbes 2020;13:1–7. 10.1080/19490976.2020.1730294 PMC7524361 32167021

